# Prosthetic rehabilitation of edentulous hemimandibulectomy patient: a clinical report

**DOI:** 10.1002/ccr3.1125

**Published:** 2017-08-15

**Authors:** Shailendra Kumar Sahu, B.K. Motwani, Anurag Dani

**Affiliations:** ^1^ Department of Prosthodontics Chhattisgarh Dental College and Research Institute Rajnandgaon Chhattisgarh 491441 India

**Keywords:** Edentulous, hemimandibulectomy, nonanatomic teeth, rehabilitation, twin rows of teeth

## Abstract

Surgical resection of mandible causes deviation of mandible toward affected side, resulting in loss of occlusal contact. This article presents twin rows of nonanatomic teeth on the untreated side of maxillary complete denture. The palatal row helps in mastication, whereas the buccal row supports the cheeks and improves the aesthetics.

## Introduction

Neoplastic lesions of the oral cavity are mostly surgically treated, which necessitates radical surgery of the tongue, floor of the mouth, and mandible 1, 2. Mandibulectomy and radical neck surgery implicate the comprehensive loss of tissues and affiliated functions. For edentulous patients, Cantor and Curtis developed a classification system based on the extent of the mandible that has been extirpated or restored 3.

In patients undergone with mandibular extirpation, the residual mandibular fragment usually retrudes and deviates to the extirpated side. On opening mouth, this diversion increases, resulting in facial asymmetry and failure of function. This is compounded if the patient is edentulous because of failure of proprioception 4, 5, 6, 7. Failure of proprioception results in coarse and indefinite movement of the mandible. This factor, with the addition of impaired tongue function, may totally compromise mastication and speech.

Review of the literature reveals various designs of prostheses that have been employed to treat edentulous hemimandibulectomy patients 4, 5, 6, 8, 9, 10, 11, 12. Lloyd described artificial temporomandibular joints to treat edentulous patient 8, but they are now discontinued. Fattore et al. rehabilitated a edentulous hemimandibulectomy patient with the help of a two‐piece Gunning splint 9. However, these are no longer used because of its several disadvantages like intermaxillary fixation, poor hygiene, and it is not useful in patients who have undergone radical neck surgery 10. Few authors have rehabilitated edentulous hemimandibulectomy patient with the aid of a palatal ramp attached to maxillary posterior teeth on the untreated side 4, 5, 6. Various authors have suggested the modification of the palatal ramp, which utilizes multiple maxillary teeth in the form of twin rows on the untreated side 10, 11, 12. However, most of these case reports in the literature deal with partially edentulous patients and very few are related to completely edentulous patients. The present case report illustrates the rehabilitation of edentulous hemimandibulectomy patient by utilizing twin rows of maxillary posterior teeth on the untreated side.

## Case Presentation

**Figure 1:**
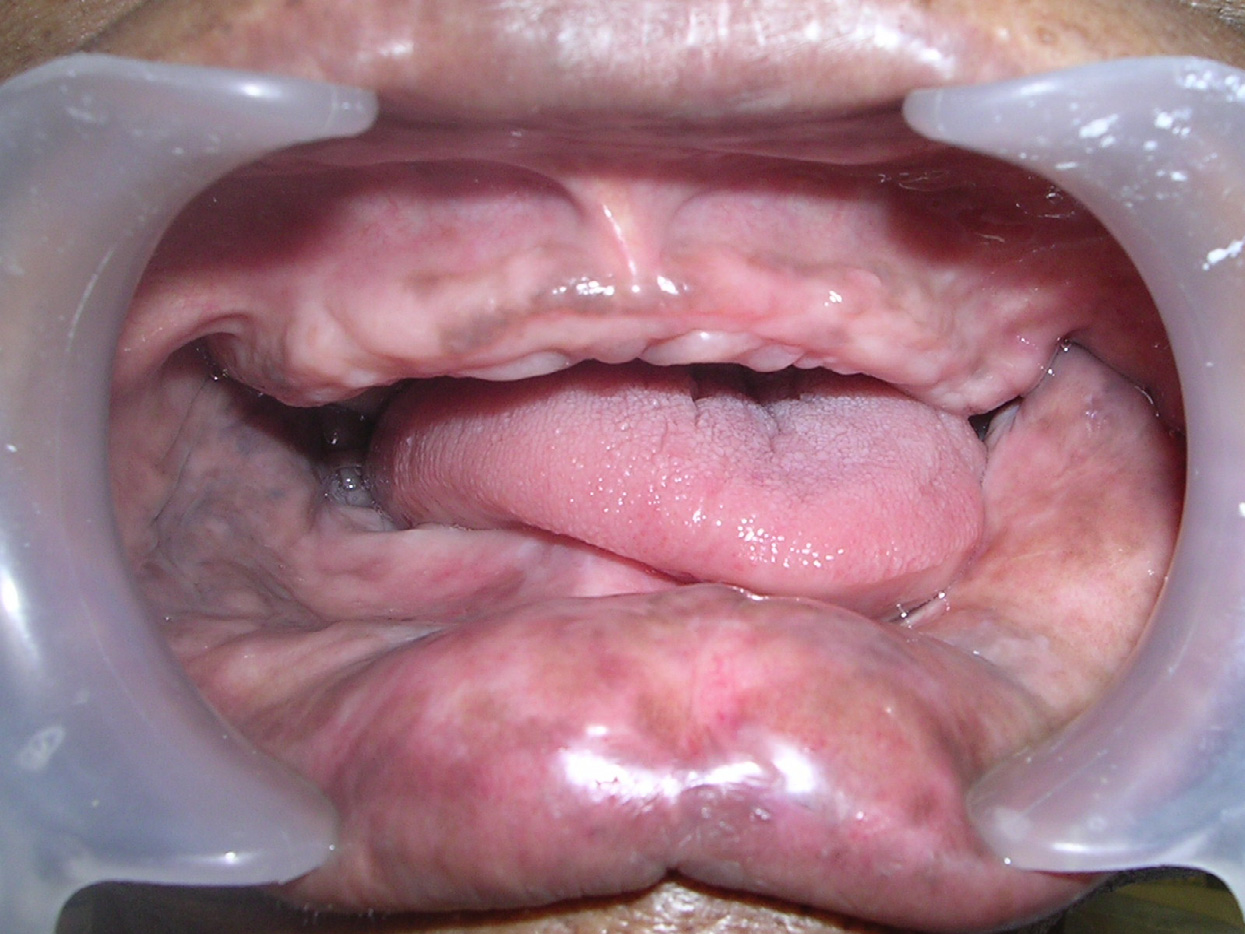
Preoperative intra‐oral view showing mandibular deviation toward resected side.

A 56‐year‐old man who had undergone hemimandibulectomy was referred to the Department of Prosthodontics, Chhattisgarh dental college . The chief complaint of patient was difficulty in mastication and speech. Patient's history revealed that he was a tobacco chewer for almost 35 years. Two years back, he was diagnosed with squamous cell carcinoma of the left buccal mucosa and alveolus. He underwent extensive resection of the mandible distal to the left lower canine region involving the ramus, coronoid process, and condyle, together with radical neck dissection about 18 months ago. Pectoralis major myocutaneous flap (PMMF) was used for reconstruction. As per the Cantor and Curtis classification, patient had Type II type of resection, that is, unilateral discontinuity mandibular defect on the left side (Fig. 1).

Primary impression was made using medium fusing impression compound with non perforated stock metal trays. The principle of broad tissue coverage within physiologic limit was utilized while making impressions. Custom trays were fabricated using acrylic resin over primary casts. The border molding of the maxillary tray was performed using low fusing modeling compound stick (DPI, Dental products of India, Mumbai, India). The putty elastomeric impression material (3M ESPE Express, Germany) was used for border molding mandibular tray, and medium‐body (3M ESPE Express, Germany) vinyl polysiloxane was used for making final impressions. Master cast so obtained was used to fabricate temporary record bases with self‐cure acrylic (DPI, Mumbai, India) resin. The palatal surface of maxillary wax rim was widened on the unresected side, because of deviation of mandible. Occlusal vertical dimension was established using phonetics, closest speaking space, and swallowing methods. Centric occlusion registration was obtained with wax by manipulating the mandible into most advantageous position that was within the reach of the patient.

**Figure 2:**
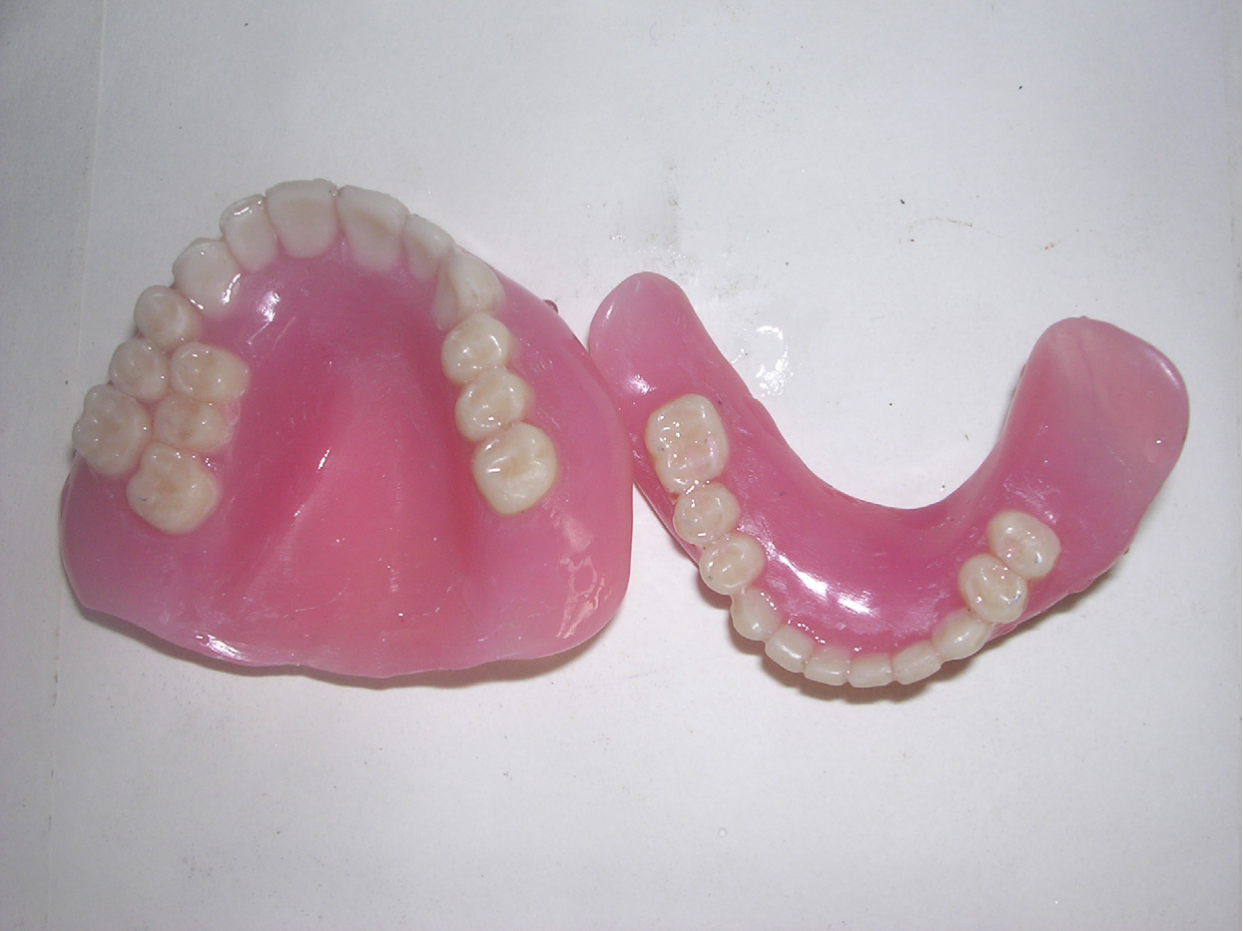
Final prosthesis.

**Figure 3:**
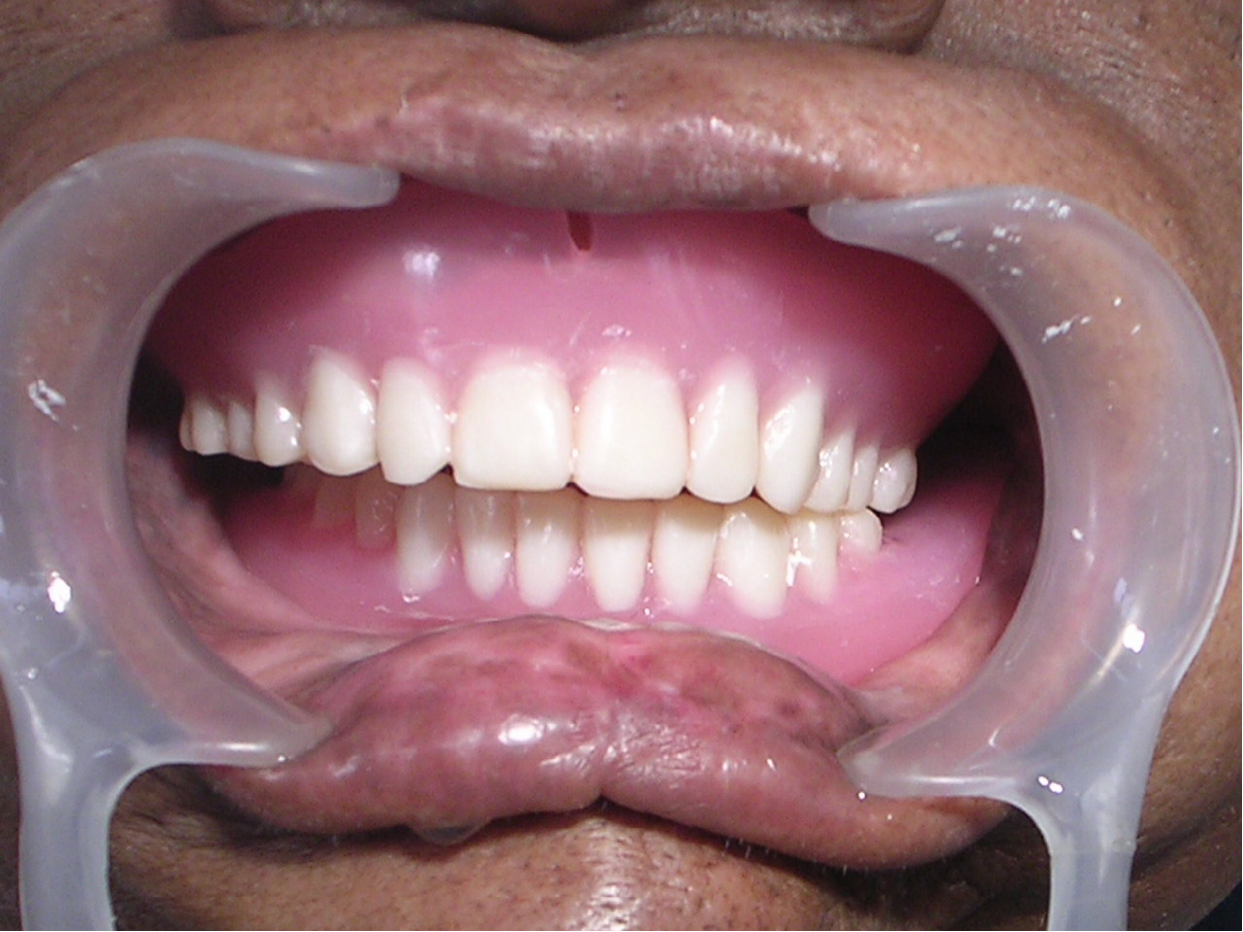
Intra‐oral view of the patient with the denture.

Nonanatomic posterior teeth were used, so that the mandible was free to move in lateral direction. The teeth were initially positioned according to the contours of the wax rim. In order to compensate the mandibular deviation, modification was performed in teeth arrangement. Maxillary anterior teeth were arranged lingual to and mandibular anterior teeth were arranged labial to their accustomed position. Similarly, the mandibular posterior teeth were initially arranged according to the contours of the wax rims. Then, the mandible premolars and molars on the untreated side were placed slightly buccal to the crest of the ridge. However, premolars and molars on the resected side were arranged lingual to the crest of the ridge. Twin rows of premolars and molars were arranged on maxillary denture on the untreated side (Fig. 2). The waxed‐up dentures were tried intra‐orally and evaluated for aesthetics, phonetics, and occlusion. The processed dentures were evaluated intra‐orally (Fig. 3), and the necessary occlusal adjustments were carried out. Conventional postinsertion instructions were told. The patient was encouraged to learn to adapt to the new dentures. Physiotherapy in the form of light exercises such as repeatedly opening and closing the mandible into occlusion was advised to the patient. This helped in improving the neuromuscular coordination. In the early days, patient was facing difficulty in manipulating the denture but with the repeated use over a period of time, patient experienced satisfactory mastication and phonetics. Six month follow‐up revealed that patient was functionally and psychologically satisfied.

## Discussion

The rehabilitation of patient after hemimandibulectomy is challenging for the prosthodontist. The surgical excision of unilateral mandible causes the mandible to deviate toward the extirpated side with the loss of occlusal contact 1. Dentulous patients can be retrained to achieve acceptable maxillomandibular relationship with the help of appliance like guide plane 1, 12, 13, 14, 15. The success of mandibular guide flange appliance depends on early placement of the appliance preferably as soon as the immediate postsurgical sequelae have subsided 15. However, these appliances cannot be used in edentulous patients. Hence, edentulous patients most of the times never achieve adequate maxillomandibular relationship which affects both mastication and aesthetics 3. The present case report illustrates a effective method of rehabilitating such patients.

The objectives of the master impression are the same as in the conventional dentures, that is, to establish retention, provide support and stability, create esthetic, and preserve the remaining tissues. Retention in the mandibular denture was achieved by obtaining close adaptation of the prosthesis with the bearing surface and by extending the lingual periphery on the unresected side to the maximum extent compatible with functional and anatomical limitations. The stability of the prosthesis was facilitated by developing the contours of the lingual flange. Peripheral seal of the maxillary denture may be difficult to achieve in some patients because with the deviation of the mandible, coronoid process and ramus may be in close apposition to the tuberosity on the nonresected side 1.

The jaw relation was difficult to establish because of mandibular retrusion and deviation to the extirpated side, poor neuromuscular coordination, and the absence of proprioceptive impulses from teeth. Evaluation with phonetics and closest speaking space is best suited for the determination of vertical dimension of rest and vertical dimension of occlusion. The more favorable maxillomandibular relationship obtained with the centric occlusion registration record, the more favorable the prosthodontic prognosis. The clinician should manipulate the mandible and place it in the most advantageous position that is within the reach of the patient 1.

The deviation of mandible on closure, lack of definitive end point of closure, the loss of proprioception, and the increased lateral stresses exerted on the denture bases during function favor the use of nonanatomic posterior teeth 4. It is advisable to place the maxillary anterior teeth lingual to and mandibular anterior teeth labial to the accustomed position because mandible deviates and retrudes on opening. The mandibular premolars and molars on the untreated side were placed slightly buccal to the crest of the ridge. However, premolars and molars on the resected side were arranged lingual to the crest of the ridge. Some authors have suggested the use of ramps on the maxillary teeth 4, 6. These ramps should be approximately 5–10 mm wide depending on the extent of the mandibular deviation. In the present case, an attempt has been made to modify the ramp by providing twin rows of teeth on the uninvolved side of the maxillary denture so as to provide freedom in movement of mandible at the established vertical dimension. The palatal row helps in mastication, whereas the buccal row supports the cheeks and improves the aesthetics.

Patients should be monitored closely during the postinsertion period. In addition, many patients require continual support and encouragement. The use of the prosthesis for the mastication should be deferred for at least a week. As the patient uses the prosthesis, some occlusal adjustment is required. Along with the prosthetic rehabilitation, physiotherapy in the form of light exercises is essential as it helps in improving neuromuscular coordination, which ultimately improves mastication and phonetics.

## Conclusion

The rehabilitation of edentulous hemimandibulectomy patients necessitates revamping of certain basic principles of conventional prosthodontics because of several compromising factors. In edentulous patients, an extended occlusal table established by providing twin rows of nonanatomic teeth on maxillary denture will aid to enhance the stability of the dentures and thus improve masticatory efficiency as well as aesthetics.

## Authorship

SSK: performed the clinical management and follow‐up of the patient, involved in the literature search, wrote the first draft, and edited all versions of manuscript. MBK: analyzed the case report, involved in the literature review, and critically evaluated the manuscript. DA: performed the follow‐up of the patient, acquisition of clinical images, and critically evaluated the manuscript.

## Conflict of Interest

None declared.
